# Supplementary material for: Genetic Mutations and Mitochondrial Redox Signaling as Modulating Factors in Hypertrophic Cardiomyopathy: A Scoping Review
Source: Int J Mol Sci. 2024 May 28;25(11):5855. doi: 10.3390/ijms25115855 (PMC11173352; doi:10.3390/ijms25115855)
Supplement: Supplementary file 1 [file ijms-25-05855-s001.zip › ijms-3001418-supplementary.pdf]

SUPPLEMENTAL

Genetic Mutations and Mitochondrial Redox Signaling as Modulating Factors in Hypertrophic Cardiomyopathy: A Scoping Review

Antonio da Silva Menezes Junior [1,*], Ana Luísa Guedes de França-e-Silva [1], Henrique Lima de Oliveira [1], Khissya Beatryz Alves de Lima [1], Iane de Oliveira Pires Porto [2], Thays Millena Alves Pedroso [2], Daniela de Melo e Silva [1] and Aguinaldo F. Freitas, Jr. [1]

[1] Faculdade de Medicina, Departamento de Clínica Médica, Universidade Federal de Goiás (UFG), Goiânia 74020-020, Brazil; ana.guedes@discente.ufg.br (A.L.G.d.F.-e.-S.); henrique.lima2@discente.ufg.br (H.L.d.O.); khissya_beatryz@discente.ufg.br (K.B.A.d.L.); danielamelosilva@ufg.br (D.d.M.e.S.); afreitasjr@msn.com (A.F.F.J.)

[2] Faculdade de Medicina, Universidade de Rio Verde (UniRV), Campus Aparecida, Aparecida de Goiânia 74345-030, Brazil; iane.porto@unirv.edu.br (I.d.O.P.P.); thays.millena04@gmail.com (T.M.A.P.)

* Correspondence: a.menezes.junior@uol.com.br

Supplemental Table S1. Baseline Characteristics of the Reviewed Articles and the Studied Population.

Author/Year	Country	Title	Type of Study	Objectives	Methods	Main findings
Dipchand et al. [1]	USA	Maternally inherited hypertrophic cardiomyopathy: A manifestation of mitochondrial DNA mutations - Clinical course in two families	Observational study	The study detailed clinical aspects and outcomes in two families with mitochondrial DNA- related hypertrophic cardiomyopathy, emphasizing variability between exclusive cardiac symptoms and typical multisystemic signs in inherited mitochondrial disorders based on mutant mitochondrial tRNA percentages.	The study outlines clinical cases from two families affected by mitochondrial DNA defects, emphasizing clinical presentation, investigations, pathological findings, and clinical progression.	The results highlighted clinical heterogeneity among affected families, revealing exclusive cardiac manifestations due to mitochondrial tRNA defects.
Sequeira et al. [5]	Germany	Mechano-energetic uncoupling in hypertrophic cardiomyopathy: Pathophysiological mechanisms and therapeutic opportunities	Review	The study aims to provide evidence from experimental and clinical studies supporting the role of hypercontractility and cellular energy alterations in the progression of HCM towards heart failure and sudden cardiac death. It emphasizes the importance of	A comprehensive review of evidence from studies exploring hypercontractility, cellular energy alterations, and other mechanisms related to the progression of HCM towards heart failure and sudden cardiac death.	Key findings emphasize hypercontractility and cellular energy alterations as central elements in the progression of HCM towards heart failure and sudden cardiac death, underscoring their relevance for therapeutic strategies.

				understanding these mechanisms for the development of effective therapeutic strategies.		
Viola et al. [8]	Austrália	Impaired calcium handling and mitochondrial metabolic dysfunction as early markers of hypertrophic cardiomyopathy	Review	The main objective is to understand the early pathophysiological mechanisms of hypertrophic cardiomyopathy (HCM), particularly related to genetic mutations in cardiac contractile proteins, aiming to identify useful markers for disease progression and inform the development of effective therapies.	A broad review of existing literature focusing on specific genetic mutations, such as those in genes associated with cardiac myosin binding protein-C, β-cardiac myosin heavy chain, cardiac troponin I, and cardiac troponin T.	Mutations in sarcomeric genes, including MYBPC3, MYH7, TNNI3, and TNNT2, contribute significantly to HCM, with early-stage similarities observed in increased calcium sensitivity and mitochondrial metabolic activity. Understanding mutation-specific pathophysiological mechanisms, particularly related to calcium handling, is crucial for developing targeted therapies, emphasizing the intricate role of calcium in HCM development.
Bhatti et al. [9]	Índia	Mitochondrial dysfunction and oxidative stress in metabolic disorders - A Step towards mitochondria based therapeutic strategies	Systematic review	The aim of this article is to highlight the recent progress regarding the mitochondrial role in metabolic syndromes and summarize the advancements of molecules targeted to mitochondria as therapeutic targets for treating metabolic syndromes.	A literature analysis was conducted on mitochondrial dysfunction and oxidative stress in metabolic disorders.	This study sheds light on disturbances in mitochondrial dynamics, oxidative stress, and metabolic dysfunctions, presenting therapeutic strategies based on mitochondria as a future perspective.
				The study aimed to investigate the		

Lee et al. [12]	USA	Cell Cycle Re-Entry and Mitochondrial Defects in MycMediated Hypertrophic Cardiomyopathy and Heart Failure	Experimental study	functional role of Myc in cardiomyopathy, demonstrating the causal relationship between sustained activation of Myc in adult cardiomyocytes and the development of HCM and congestive HF, highlighting Myc as a critical regulator of associated pathological consequences.	The study employed bitransgenic mice to selectively induce Myc expression in cardiomyocytes, addressing associated cellular, molecular, and mitochondrial mechanisms.	Sustained activation of Myc in cardiomyocytes induced severe hypertrophic cardiomyopathy, ventricular dysfunction, and congestive heart failure, highlighting Myc as a crucial regulator of these pathological outcomes.
Christiansen et al. [13]	Denmark	Impaired mitochondrial oxidative phosphorylation and fatty acid oxidation with enhanced mitochondrial oxidative stress in spontaneously-occurring feline hypertrophic cardiomyopathy	Observational study	The study aimed to understand how mitochondrial dysfunction and oxidative stress impact hypertrophic cardiomyopathy (HCM) in cats by comparing healthy cardiac and skeletal muscle tissues with those affected by HCM.	The study assessed hypertrophic cardiomyopathy (HCM) in cats, evaluating mitochondrial function in heart and skeletal muscles using high- resolution respirometry and spectrofluorometry, highlighting decreased heart mitochondrial capacity and elevated oxidative stress in HCM-affected cats compared to controls.	The results indicated that cats with hypertrophic cardiomyopathy (HCM) had reduced mitochondrial capacity in the heart and increased oxidative stress compared to healthy cats. This suggests an association between mitochondrial dysfunction and HCM development.
Li et al. [14]	China	Mitochondrial Dysfunctions Contribute to Hypertrophic Cardiomyopathy in Patient iPSC-Derived Cardiomyocytes with MT-RNR2 Mutation	Experimental study	This study aims to understand the molecular mechanisms involved in the pathology of hypertrophic cardiomyopathy caused by the	This study employed induced pluripotent stem cells from human patients with HCM and the m.2336T>C mutation.	This mutation results in mitochondrial dysfunction, structural defects, decreased levels of mitochondrial membrane potential, and increased intracellular calcium.

				m.2336T>C mutation.		
Van Der Velden et al. [15]	Netherlands	Metabolic changes in hypertrophic cardiomyopathies: scientific update from the Working Group of Myocardial Function of the European Society of Cardiology	Systematic review	This study aims to provide an overview of recent insights into metabolic changes in genetic hypertrophic cardiomyopathy and discuss therapies that can be explored to target disturbed metabolism and prevent the onset of this disease.	A literature analysis was conducted on the impacts of metabolic alterations in hypertrophic cardiomyopathy.	Although the final clinical phenotype of HCM may be indeterminate, depending on the genotype, initial mutation- induced defects in sarcomere function and subsequent changes in signaling pathways can signal significant differences based on the affected gene and even on the specific mutation.
Chen et al. [16]	China	Deletion of Gtpbp3 in zebrafish revealed the hypertrophic cardiomyopathy manifested by aberrant mitochondrial tRNA metabolism	Experimental study	Understanding the role of defective nucleotide modifiers of tRNAs in mitochondrial biogenesis and their pathological consequences in HCM.	The study employed CRISPR/Cas9 system to generate gtpbp3 knockout zebrafish	The aberrant mtRNA metabolism impaired mitochondrial respiration, induced oxidative stress, and altered respiratory chain activities, resulting in developmental changes and fractional shortening of the mutant zebrafish ventricles.
Li et al. [17]	China	MLP-deficient human pluripotent stem cell derived cardiomyocytes develop hypertrophic cardiomyopathy and heart failure phenotypes due to abnormal calcium handling	Experimental study	The study aims to generate an MLP-deficient cellular model using hESCs, investigate the pathological mechanisms underlying MLP mutations in cardiomyocytes, assess responses to pharmacological treatments and propose therapeutic strategies for MLP	The study employed CRISPR/Cas9 to generate human embryonic stem cells (hESC) without MLP, investigating their effects on cardiomyocytes.	Treatment with verapamil prevented the development of HCM and HF phenotypes, emphasizing the importance of intracellular calcium homeostasis as a potential therapeutic target.

				deficiency-associated cardiomyopathy.		
Saoura et al. [18]	USA	Mutations in ELAC2 associated with hypertrophic cardiomyopathy impair mitochondrial tRNA 3′-end processing	Experimental study	The main aim of the study was to correlate specific variants in the ELAC2 gene with mitochondrial disorders and CMH, exploring the underlying molecular mechanisms of this clinical condition.	Complete exome sequencing of patients was performed to identify mutations in ELAC2 for the in vitro study.	This study confirms the correlation of ELAC2 variants with severe early forms of hypertrophic cardiomyopathy (CMH) and mitochondrial dysfunction.
Ranjbarvaziri et al. [19]	USA	Altered Cardiac Energetics and Mitochondrial Dysfunction in Hypertrophic Cardiomyopathy	Case-control	Investigate alterations in biochemical, ultrastructural, and functional pathways related to cardiac energetic features in HCM, aiming to identify pathophysiological mechanisms with potential therapeutic targets.	Comprehensive multi- omic analysis of the molecular, ultrastructural, and functional components of cardiac energetic features in myocardial samples from 27 patients with HCM and 13 normal controls (donor hearts).	The main findings revealed metabolic changes and mitochondrial dysfunction in patients with HCM, highlighting potential therapeutic targets to enhance metabolic function and alleviate mitochondrial damage in the disease.
Zhang et al. [20]	China	Ablation of Mto1 in zebrafish exhibited hypertrophic cardiomyopathy manifested by mitochondrion RNA maturation deficiency	Experimental study	Understanding the role of Mto1 ablation in mitochondrial biogenesis and its pathological consequences in HCM.	The study employed CRISPR/Cas9 system to generate ablation of Mto1 in zebrafish potential role of these alterations in the pathophysiology of HCM.	The mutation impaired mitochondrial trans-lation and reduced activities of oxidative phosphory-lation complexes. These mitochondrial dysfunction caused heart development defects and hypertrophy of cardiomyocytes and myocardial fiber disarray in ventricles.

Previs et al. [21]	USA	Defects in the Proteome and Metabolome in Human Hypertrophic Cardiomyopathy	Observational study	The aim of this study is to examine the metabolic reprogramming in human HCM and its potential consequences on contractile function.	Metabolomic, proteomic, and targeted quantitative analyses were performed on cardiac tissue from patients with HCM.	The study identified an increase in extracellular matrix and intermediate filament proteins, along with a decrease in muscle creatine kinase and mitochondrial proteins involved in fatty acid oxidation, alongside a reduction in ATP levels present in hearts affected by HCM.
Moore et al. [22]	USA	Multi-Omics Profiling of Hypertrophic Cardiomyopathy Reveals Altered Mechanisms in Mitochondrial Dynamics and Excitation–Contraction Coupling	Experimental study	The objective of this study is to understand the initial events and direct impacts of mutations in the myosin heavy chain in cardiac cells with HCM.	This study utilized induced pluripotent stem cell-derived cardiomyocytes from patients with specific mutations associated with HCM. These were compared with cardiac cells from patients at different stages of the disease, obtained through myectomies.	This study elucidated molecular mechanisms explaining mitochondrial homeostasis in both early and advanced models of HCM.
Nollet et al. t [23]	Netherlands	Mitochondrial dysfunction in human hypertrophic cardiomyopathy is linked to cardiomyocyte architecture disruption and corrected by improving NADH- driven mitochondrial respiration.	Cross- sectional study	The study analyzed mitochondrial dysfunction and cellular architecture in hypertrophic cardiomyopathy (HCM) patients. It aimed to link mitochondrial issues, organization concerning septal thickness, and genetic mutations related to HCM. Additionally, it investigated	Cardiac tissue from hypertrophic cardiomyopathy (HCM) patients was used to analyze mitochondrial function and structure. Patients' genetic mutations and septal thickness were categorized, and interventions targeting mitochondria aimed to improve respiratory function.	Hypertrophic cardiomyopathy (HCM) patients lacking genetic mutations (genotype-negative) and with increased septal thickness showed severe mitochondrial dysfunction, correlated with disorganized mitochondria. Interventions targeting mitochondria improved respiratory function, highlighting their therapeutic potential in HCM.

				mitochondrial-targeted therapies to improve respiratory function, identifying their potential as a therapeutic focus in HCM.		
Marin- Garcia et al. [24]	USA	Cardiac mitochondrial dysfunction and DNA depletion in children with hypertrophic cardiomyopathy.	Case report	Explored mitochondrial enzyme issues in severe HCM cases, noting specific respiratory enzymes, reduced DNA, and cytochrome c oxidase subunit 2 levels.<br>Absence of known mutations suggests frequent enzyme defects. DNA depletion in one case needs more research on its role in HCM.	Explored four severe HCM cases' mitochondrial enzyme activity (complexes I, III, IV, V) and DNA<br>mutations. Studied DNA depletion and enzyme subunit levels to identify HCM-related mitochondrial issues..	The HCM study identified cardiac mitochondrial enzyme irregularities in four cases, including DNA depletion in one and reduced enzyme levels in another. These findings suggest unique mitochondrial irregularities in HCM, requiring further investigation..
Zhuang et al. [40]	China	1-Deoxynojirimycin promotes cardiac function and rescues mitochondrial cristae in mitochondrial hypertrophic cardiomyopathy.	Experimental study	Assessing the efficacy of the compound 1-Deoxynojirimycin (DNJ) in treating mitochondrial hypertrophic cardiomyopathy (HCM).	The study employed mitochondrial compound screening, assessment in hypertrophic cardiomyopathy (HCM) patient-derived cardiac cells, testing of the compound 1-Deoxynojirimycin (DNJ), and validation in an animal model to investigate its therapeutic potential for HCM.	This study concludes that the compound 1-Deoxynojirimycin (DNJ) proved effective in restoring compromised mitochondrial function in mitochondrial hypertrophic cardiomyopathy (HCM). DNJ facilitated mitochondrial cristae reconstruction, improved calcium homeostasis, and normalized electrophysiological properties in HCM-affected cardiac cells.<br>Moreover, DNJ

						demonstrated efficacy in reducing cardiac hypertrophy in an angiotensin II-induced hypertrophy animal model. These findings suggest the potential of DNJ as a promising therapeutic agent for mitochondrial HCM, paving the way for novel therapeutic approaches in this condition.
Li et al. [58]	China	Mitochondrial dysfunction caused by m.2336T>C mutation with hypertrophic cardiomyopathy in cybrid cell lines.	Experimental study	To elucidate the molecular mechanisms by which the m.2336T>C mutation in mitochondrial DNA contributes to the development of hypertrophic cardiomyopathy (HCM). Researchers used cell lines to investigate the direct effects of this mutation on 16S ribosomal RNA stability, mitochondrial translation, reactive oxygen species (ROS) generation, ATP production, mitochondrial membrane potential, and cell survival.	The researchers used cell lines to create experimental models, generating cybrids. They performed various functional assays, analyzing mitochondrial RNA stability, translation, ROS production, ATP levels, mitochondrial membrane potential, and cell survival to understand the mutation's direct impact on mitochondrial function and cell physiology.	The m.2336T>C mutation led to mitochondrial dysfunction, evidenced by unstable 16S ribosomal RNA, reduced mitochondrial translation, increased ROS generation, decreased ATP production, impaired mitochondrial membrane potential, and decreased cell survival. These findings suggest the mutation's role in hypertrophic cardiomyopathy (HCM).
Vakrou et al. [53]	USA	Hypertrophic cardiomyopathy: a heart in need of an energy bar?	Systematic review	The primary goals of this review are to explore the relationship between cellular metabolism, mitochondrial function, and hypertrophic cardiomyopathy (HCM). It aims to elucidate how mutations in sarcomeric proteins lead to variable	A literature review was conducted on the impacts of mitochondrial metabolism in hypertrophic cardiomyopathy.	According to this study, abnormalities in calcium cycling, oxidative stress, mitochondrial dysfunction, and energy deficiency play a significant role in HCM pathology, with cellular and mitochondrial metabolism being a pathway for future therapies.

				cardiac phenotypes in individuals affected by HCM. Additionally, the review intends to highlight the role of abnormalities in calcium cycling, oxidative stress, mitochondrial dysfunction, and energetic deficiency in the pathophysiology of HCM based on existing evidence.		
Nollet et al. [54]	Germany	Western diet triggers cardiac dysfunction in a heterozygous Mybpc3-targeted knock-in hypertrophic cardiomyopathy mouse model	Experimental study	The objectives were to investigate how a Western diet influences the development of hypertrophic cardiomyopathy in genetically modified mice, examining the metabolic, molecular, and cardiac changes associated with this condition.	The study involved feeding Western diet to genetically modified mice to mimic the effects of metabolic syndrome and prediabetes. Cardiac function, metabolism, and molecular analyses were conducted to understand the onset of cardiac disease in hypertrophic cardiomyopathy.	The results showed that feeding a Western diet triggered cardiac dysfunction and hypertrophy in mice with the Mybpc3c.772G>A mutation, suggesting a link between the mutation- induced increase in sarcomeric ATP consumption and metabolic inflexibility in the heart under obesity- related risk factors.
Chen et al. [55]	China	17β-estradiol prevents cardiac diastolic dysfunction by stimulating mitochondrial function: A preclinical study in a mouse model of a human hypertrophic cardiomyopathy mutation	Experimental study	Investigate the effect of ovariectomy (OVX) and 17 b-estradiol (E2) replacement on both mitochondrial and myocardial function in cTnT-Q92 transgenic mice generated by cardiac-restricted expression of a human hypertrophic cardiomyopathy (HCM) mutation	The study involved transgenic mice with hypertrophic cardiomyopathy, subjected to ovariectomy and estradiol treatment, analyzing cardiac function, mitochondrial metabolism, and protein expression.	Estradiol improved cardiac function, stabilized energy metabolism, and reduced oxidative stress in mice with hypertrophic cardiomyopathy.
				To investigate whether mitochondrial		Mice with HCM-related mutations showed

Lucas et al. [62]	USA	Alterations in mitochondrial function in a mouse model of hypertrophic cardiomyopathy.	Experimental study	dysfunction contributes to the variability of hypertrophic cardiomyopathy (HCM) phenotypes in transgenic mouse models. The objectives were to understand how mitochondrial alterations could impact cardiac function and relate to the heterogeneity of observed HCM phenotypes.	Transgenic mice with HCM mutations were studied to assess cardiac involvement in mitochondrial dysfunction.	reduced mitochondrial respiration and enzyme activity, alongside structural abnormalities. These findings emphasize the critical role of mitochondrial dysfunction in HCM and suggest a potential link to its diverse phenotypes".
Christiansen et al. [67]	Denmark	Ultrastructural myocardial changes in seven cats with spontaneous hypertrophic cardiomyopathy	Experimental study	Investigate the ultrastructural changes in the myocardium of cats diagnosed with hypertrophic cardiomyopathy.	Biopsies from the myocardium of seven cats diagnosed with hypertrophic cardiomyopathy and eight control cats were collected and analyzed using transmission electron microscopy to identify ultrastructural changes, particularly in the cytoskeleton and mitochondria.	The key findings highlight ultrastructural changes in the myocardium of cats with hypertrophic cardiomyopathy, particularly in the cytoskeleton and mitochondria, underscoring the importance of subsequent investigations to elucidate the underlying pathogenic mechanisms of this condition.
Liu et al. [73]	China	Ablation of ALCAT1 mitigates hypertrophic cardiomyopathy through effects on oxidative stress and mitophagy.	Experimental	To investigate the role of the ALCAT1 enzyme in mitochondrial dysfunction associated with hypertrophic cardiomyopathy and comprehend its potential as a link between oxidative stress and mitochondrial deterioration in age-	Mice with targeted deletion of the ALCAT1 gene were generated to assess the impact of its expression on HCM.	Increased expression of the ALCAT1 enzyme in cardiac cells resulted in oxidative stress, lipid peroxidation, and reduced mitochondrial DNA. Conversely, the absence of ALCAT1 prevented T4- induced cardiomyopathy

			study	related heart diseases. The researchers aimed to examine the effects of ALCAT1 expression and absence in cardiac cells, assessing their relationship with oxidative stress, lipid peroxidation, mitochondrial DNA depletion, and their influence on cardiac and metabolic function.		development and associated cardiac dysfunction. This suggests that ALCAT1 may serve as a crucial link between oxidative stress and mitochondrial dysfunction in age-related heart diseases.
Chouchani et al. [74]	UK	Complex I deficiency due to selective loss of Ndufs4 in the mouse heart results in severe hypertrophic cardiomyopathy	Experimental study	The study aimed to investigate whether the reduction of mitochondrial complex I activity in the heart alone is sufficient to induce hypertrophic cardiomyopathy, irrespective of increased mitochondrial hydrogen peroxide levels or oxidative damage.	The study used heart-specific genetic ablation (Ndufs4-null mice) to induce a chronic 50% reduction in mitochondrial complex I activity, assessing hypertrophic cardiomyopathy development via specific heart tests and MRI.	Ndufs4-null mice with reduced heart mitochondrial complex I activity (<50%) developed severe hypertrophic cardiomyopathy, yet without notable increases in mitochondrial hydrogen peroxide or oxidative damage markers, indicating the sufficiency of reduced complex I activity alone in triggering cardiomyopathy independently of heightened oxidative stress.
		Myocardial		The main goals were to investigate, using a computational model (EMME), how hypertrophic cardiomyopathy (HCM) impacts cellular and	An EMME model replicated human hypertrophic cardiomyopathy by modifying ion channels and	The study's key findings revealed that the EMME model accurately replicated observed kinetic properties of hypertrophic cardiomyopathy (HCM). It highlighted

Adeniran et al. [75]	UK	electrophysiological, contractile and metabolic properties of hypertrophic cardiomyopathy: Insights from modelling	Computational study	organ-level electro-physiological activity, contractility, and energy metabolism regulation.<br>The study focused on understanding the molecular changes' effects caused by HCM, particularly in ion channels, calcium handling proteins, and calcium sensitivity of myofibrillar proteins.	Ca2+ handling proteins, unveiling compromised energy metabolism and altered contractile efficiency in a 3D model of the human left ventricle with septal hypertrophy.	compromised energy metabolism, evidenced by a decreased phosphocreatine to ATP ratio, and showcased altered contractile efficiency in a 3D human left ventricle model with septal hypertrophy. These results offer crucial insights into HCM's impact on cellular function, shedding light on potential pathways affecting energy regulation and heart contractility.
Wang et al. [79]	China	Case report: Rare novel MIPEP compound heterozygous variants presenting with hypertrophic cardiomyopathy, severe lactic acidosis and hypotonia in a Chinese infant	Case report	Identify rare genetic mutations causing severe conditions like hypertrophic cardiomyopathy, lactic acidosis, and hypotonia in an 8-month-old, focusing on discovering new variants in the MIPEP gene linked to combined oxidative phosphorylation deficiency-31 (COXPD31).	The study used trio whole-exome sequencing (WES) and copy number variation sequencing to identify mutated genetic loci. Validation of single nucleotide variants was done through Sanger sequencing, while quantitative real-time PCR validated copy number variants.	The 8-month-old boy presented with hypertrophic cardiomyopathy, severe lactic acidosis, and hypotonia. Genetic analysis revealed novel compound heterozygous variants in the MIPEP gene inherited from each parent. Additionally, a significant reduction in mitochondrial DNA copy number was observed.<br>These findings expanded the understanding of MIPEP mutations and provided insights into COXPD31 genotype-phenotype correlations.
				The objectives were to assess mitochondrial function in hypertrophic	The study used respiratory measurements and electron microscopy on	

Nollet et al. [80]	Germany	"Disentangling" mitochondrial dysfunction in hypertrophic cardiomyopathy	Observational study	cardiomyopathy (HCM) patients, identify mitochondrial dysfunction-related changes, and explore approaches to improve cardiac function by targeting mitochondria as a therapeutic target in HCM.	septal tissue from HCM patients to evaluate oxidative phosphorylation pathways and mitochondrial structure. It also tested drug interventions targeting mitochondrial function improvement ex vivo.	Mitochondrial function varied in HCM patients, especially those with thicker hearts, showcasing potential targeting of mitochondria for therapeutic interventions in severe cases.
Cibi et al. [82]	Singapore	Prdm16 Deficiency Leads to Age-Dependent Cardiac Hypertrophy, Adverse Remodeling, Mitochondrial Dysfunction, and Heart Failure.	Experimental study	To understand the role of the Prdm16 protein in the heart, particularly its influence on preventing cardiac hypertrophy and preserving age-related cardiac function. Additionally, the study aimed to investigate how the absence of Prdm16 affects mitochondrial function, regulation of genes associated with pathological hypertrophy, and susceptibility to heart failure in response to metabolic stress.	Researchers deleted Prdm16 in mouse hearts to study its impact. They assessed cardiac function, mitochondrial function, gene expression related to heart enlargement, and responses to metabolic stress using molecular techniques. They examined the effects on both young and aged mice to understand Prdm16's role in age- related heart issues.	The absence of Prdm16 resulted in cardiac hypertrophy, excessive ventricular fibrosis, mitochondrial dysfunction, and heart failure. Prdm16, along with euchromatic histone-lysine N-methyltransferase factors, inhibited the pro-hypertrophic transcription factor Myc, reducing the expression of fetal genes reactivated in pathological hypertrophy. Young mice lacking Prdm16 exhibited normal cardiac function but were susceptible to heart failure under metabolic stress, highlighting Prdm16's protective role against age-related cardiac hypertrophy and heart failure.
Li et al.	Taiwan	Structural and biochemical evidence of mitochondrial depletion in pigs with hypertrophic	Observational study	The main objectives of this study were to investigate mitochondrial deficiencies and the quantity of mitochondrial DNA in pig hearts affected by hypertrophic cardiomyopathy (HCM), aiming to understand the potential relationship	Pig hearts with naturally occurring HCM were utilized, employing Southern blot analysis and PCR.	Pig hearts affected by hypertrophic cardiomyopathy exhibited decreased mitochondrial enzyme activities and reduced mitochondrial DNA content compared to healthy hearts. These differences correlated

[84]		cardiomyopathy.		between these deficiencies and the development of HCM.		with the severity of cardiac hypertrophy, suggesting a potential link between mitochondrial deficiencies and the development of hypertrophic cardiomyopathy.

HCM:hypertrophic cardiomyopathy;**MLP:**Muscle LIM Protein;**OVX:**ovariectomy;**E2:** 17 b-estradiol;**hESCs:**human Embryonic Stem Cells;**HF:**Heart Failure; **tRNA:**transfer RNA; **CRISPR/Cas9:**Clustered Regularly Interspaced Short Palindromic Repeats;**MRI**:Magnetic Resonance Imaging;**MIPEP:**Mitochondrial Intermediate Peptidase;**COXPD31:**oxidative phosphorylation deficiency-31**;PCR:**Polymerase Chain Reaction;**WES:**whole-exome sequencing;**EMME:**Electro-Mechanical, Mitochondrial Energetics; **ATP:**Adenosine Triphosphate; **NADH:**Nicotinamide Adenine Dinucleotide; **PRDM16:**PR Domain Containing 16; **ALCAT1:**Acyl-CoA:lysocardiolipin acyltransferase-1; **DNA:**Deoxyribonucleic Acid.
